# Discovery and characterization of potent IL-21 neutralizing antibodies via a novel alternating antigen immunization and humanization strategy

**DOI:** 10.1371/journal.pone.0211236

**Published:** 2019-01-25

**Authors:** Reena Varkey, Qun Du, Jodi L. Karnell, Xiaodong Xiao, Kerry A. Casey, Rob Woods, Kim Rosenthal, Susan Wilson, William F. Dall’Acqua, Herren Wu, Ronald Herbst, Rachel Ettinger, Melissa Damschroder

**Affiliations:** 1 Department of Antibody Discovery and Protein Engineering, MedImmune LLC, Gaithersburg, Maryland, United States of America; 2 Department of Respiratory, Inflammation, and Autoimmunity, MedImmune LLC, Gaithersburg, Maryland, United States of America; Emory University School of Medicine, UNITED STATES

## Abstract

Interleukin-21 (IL-21), a member of the common cytokine receptor γ chain (γ_c_) family, is secreted by CD4^+^ T cells and natural killer T cells and induces effector function through interactions with the IL-21 receptor (IL-21R)/γ_c_ complex expressed on both immune and non-immune cells. Numerous studies suggest that IL-21 plays a significant role in autoimmune disorders. Therapeutic intervention to disrupt the IL-21/IL-21R/γ_c_ interaction and inhibit subsequent downstream signal transduction could offer a treatment paradigm for these diseases. Potent neutralizing antibodies reported in the literature were generated after extensive immunizations with human IL-21 alone and in combination with various adjuvants. To circumvent the laborious method of antibody generation while targeting a conserved functional epitope, we designed a novel alternating-antigen immunization strategy utilizing both human and cynomolgus monkey (cyno) IL-21. Despite the high degree of homology between human and cyno IL-21, our alternating-immunization strategy elicited higher antibody titers and more potent neutralizing hybridomas in mice than did the immunization with human IL-21 antigen alone. The lead hybridoma clone was humanized by grafting the murine complementarity-determining regions onto human germline framework templates, using a unique rational design. The final humanized and engineered antibody, MEDI7169, encodes only one murine residue at the variable heavy/light-chain interface, retains the sub-picomolar affinity for IL-21, specifically inhibits IL-21/IL-21R–mediated signaling events and is currently under clinical development as a potential therapeutic agent for autoimmune diseases. This study provides experimental evidence of the immune system’s potential to recognize and respond to shared epitopes of antigens from distinct species, and presents a generally applicable, novel method for the rapid generation of exceptional therapeutic antibodies using the hybridoma platform.

## Introduction

Interleukin-21 (IL-21) belongs to a family of immune modulatory cytokines that includes IL-2, IL-4, IL-7, IL-9, and IL-15 and has a wide range of biologic activities. IL-21 signaling takes place via a receptor complex consisting of its own unique receptor, the IL-21R, and the common gamma receptor chain (γ_c_), leading to the activation of the Janus-activated kinases (JAK) and the signal transducer and activator of transcription (STAT) pathways [1, 2]. IL-21 is mainly produced by activated CD4^+^ T cells and natural killer (NK) T cells, whereas IL-21R is expressed on a broad array of cell types, including hematopoietic and nonhematopoietic cells [3–5]. IL-21 modulates various aspects of immune function, including differentiation of CD4^+^ T cells and B cells and upregulation of CD8^+^ T-cell and NK-cell cytolytic activity. The most profound impact of IL-21 is its ability to shape the humoral immune response. IL-21 has wide-reaching actions in determining how B cells respond to their environment, as well as the potential to induce robust B-cell activation, class switch recombination, and plasma cell (PC) differentiation in concert with CD40 engagement [6].

Overexpression of IL-21 is a feature of many inflammatory and autoimmune disorders, including Sjögren’s syndrome, systemic lupus erythematosus, type 1 diabetes, multiple sclerosis, rheumatoid arthritis, and inflammatory bowel disease [7–14]. The critical role of IL-21 in promoting humoral and cellular immune responses makes it an important focus of potential therapeutic interventions in conditions characterized by both overproduction of IL-21 and pathogenic autoantibodies. Disruption of IL-21/IL-21R–mediated cell signaling has been investigated for disease control through the generation of antibodies directly targeting IL-21 [15], or IL-21R [16, 17] or the use of IL-21R fragment crystallizable (Fc) fusion protein (IL-21R-Fc) [18, 19]. The binding affinity of human IL-21 to its receptor is reported to be 70 pM [20] which makes the generation of inhibitory antibodies extremely challenging.

Several platforms have been employed to expedite the production of antibodies for research, diagnostic, and therapeutic applications [21]. Although each method has its unique potential, the hybridoma platform continues to be widely used to generate monoclonal antibodies (mAbs) [22, 23]. Of the therapeutic antibodies marketed in 2016 in the United States, only 6 have been derived from the phage display platform, whereas all others trace their origin to the hybridoma platform [24, 25]. One of the major advantages of hybridoma technology is the ability to isolate high-affinity antibodies, as immunizations can be performed in a natural biological setting that allows for the *in vivo* affinity maturation process. Human immunoglobulin G (IgG) transgenic mice have previously been used to generate IL-21–neutralizing antibodies via a complex immunization and screening process [15]. We sought to determine whether a rationally designed immunization protocol could simplify and significantly improve the anti-IL-21–specific response in mice. This question is based on our hypothesis that immune responses can be modulated to converge on specific, implicit epitopes when antigens that share the conserved, functional regions are administered in an alternating fashion during immunization. Sequential immunization with various mutant versions of the same antigen [26], or mixing multiple antigens together during immunization [27] have previously been reported. Here we report a unique immunization method alternating human and cyno IL-21 to elicit cross reactive antibodies. Cyno IL-21 was selected due to its high sequence homology to human IL-21 and highly conserved IL-21R binding region. To our knowledge, systematically alternating versions of the same antigen from different species to raise cross-reactive neutralizing antibodies has not been previously described. We therefore set out to use IL-21 as a test case to investigate such a hypothesis, which has important theoretical and practical implications in the field of immunology as well as for the improvement of hybridoma platforms.

Despite the high affinity and potency of murine antibodies generated by the hybridoma platform, their clinical usage is significantly limited due to the human anti-mouse antibody (HAMA) response which prevents repeated administration in patients [28]. In an effort to minimize their potential immunogenicity in the clinical setting, several approaches have been described to humanize the non-human antibodies intended for development as therapeutic reagents [29, 30]. The most common of these approaches is complementarity-determining region (CDR) grafting, in which murine CDRs are transferred onto a human acceptor framework (FR) derived from either a germline or a mature human antibody sequence. However, this process often results in reduced binding to the target, subsequently requiring back-mutations to murine FR residues to recover the affinity, and potentially involving extensive antibody engineering efforts [31]. In this study, we also describe the humanization and engineering of the lead anti–IL-21 hybridoma with a unique, rationally designed CDR grafting method that resulted in efficient humanization of the molecule while maintaining its high affinity and potency. Combining this novel alternating-antigen immunization strategy and rapid humanization approach yielded an IL-21 neutralizing antibody that is currently under clinical development for the treatment of Systemic Lupus Erythematosus (SLE).

## Results

### Neutralizing hybridomas elicited by alternating-antigen immunization

IL-21 represents an ideal target to test the hypothesis that an alternating-immunization strategy will yield cross-reactive neutralizing antibodies. Despite the high sequence homology between human and cyno IL-21 (95.5% identical) [32] and cross-species interactions, the binding profiles of human and cyno IL-21 to hIL-21R-Fc were different (S1 and S2 Figs). This observation suggests that discrete differences in the binding epitopes on human and cyno IL-21 could be responsible for the divergent interactions with human IL-21R. Indeed, structural analysis of the IL-21/IL-21R complex [33] showed that six residues differ between the two species: five variations occurring at sites distal to the receptor/cytokine interaction axis, and one mismatched amino acid (V91L) within the receptor binding region of IL21, suggesting a single residue could putatively be responsible for the reduced binding and activation of human IL-21R by cyno IL-21. This divergence in sequence may thus be exploited, focusing the immune response to the most functional, conserved domains (receptor binding sites) that encompass the single amino acid difference by alternating human and cyno IL-21 as immunogens. As an added benefit, the use of cyno IL-21 enables the identification of cross reacting neutralizing antibodies that facilitate *in vivo* preclinical characterization and safety assessment of this therapeutic.

**Fig 1:**
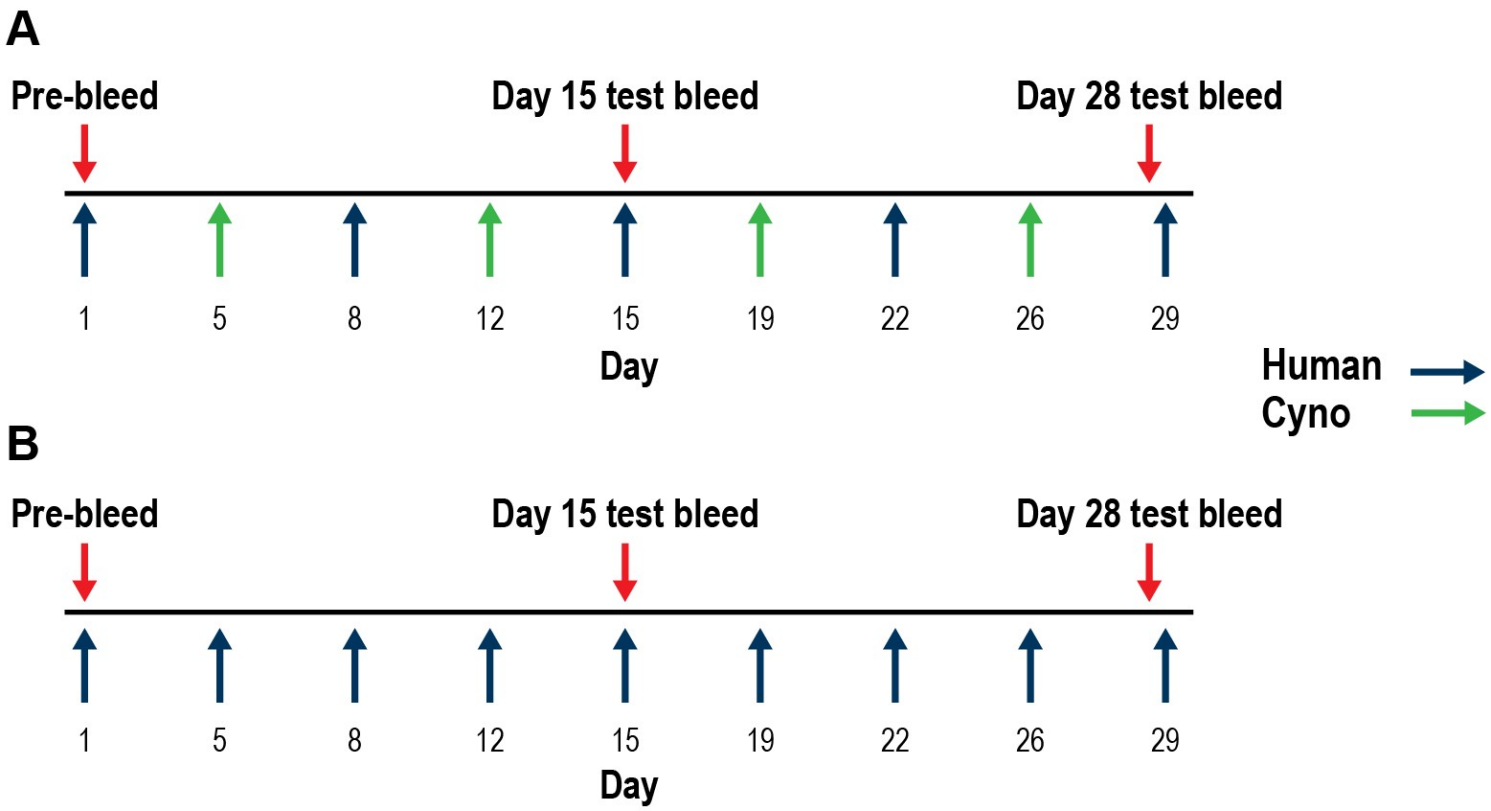
Alternating dual-antigen vs single-antigen immunization strategy for inducing a potent neutralizing antibody response in mice. (A) Alternating immunization of mice with human IL-21–BSA and cyno IL-21–BSA in alum adjuvant via the intraperitoneal and metatarsal routes. (B) Immunization of mice with human IL-21–BSA in alum adjuvant via the intraperitoneal and metatarsal routes.

Two parallel immunization campaigns were initiated: a single-antigen protocol, using only human IL-21 with bovine serum albumin (BSA) as an immunogen, and a novel dual-antigen protocol, using alternating recombinant human IL-21 with BSA and cyno IL-21 with BSA as immunogens (Fig 1). Mice sera were screened for human and cyno IL-21 binding as well as for inhibition of IL-21/IL-21R-Fc interactions in a competition enzyme-linked immunosorbent assay (ELISA).

**Fig 2:**
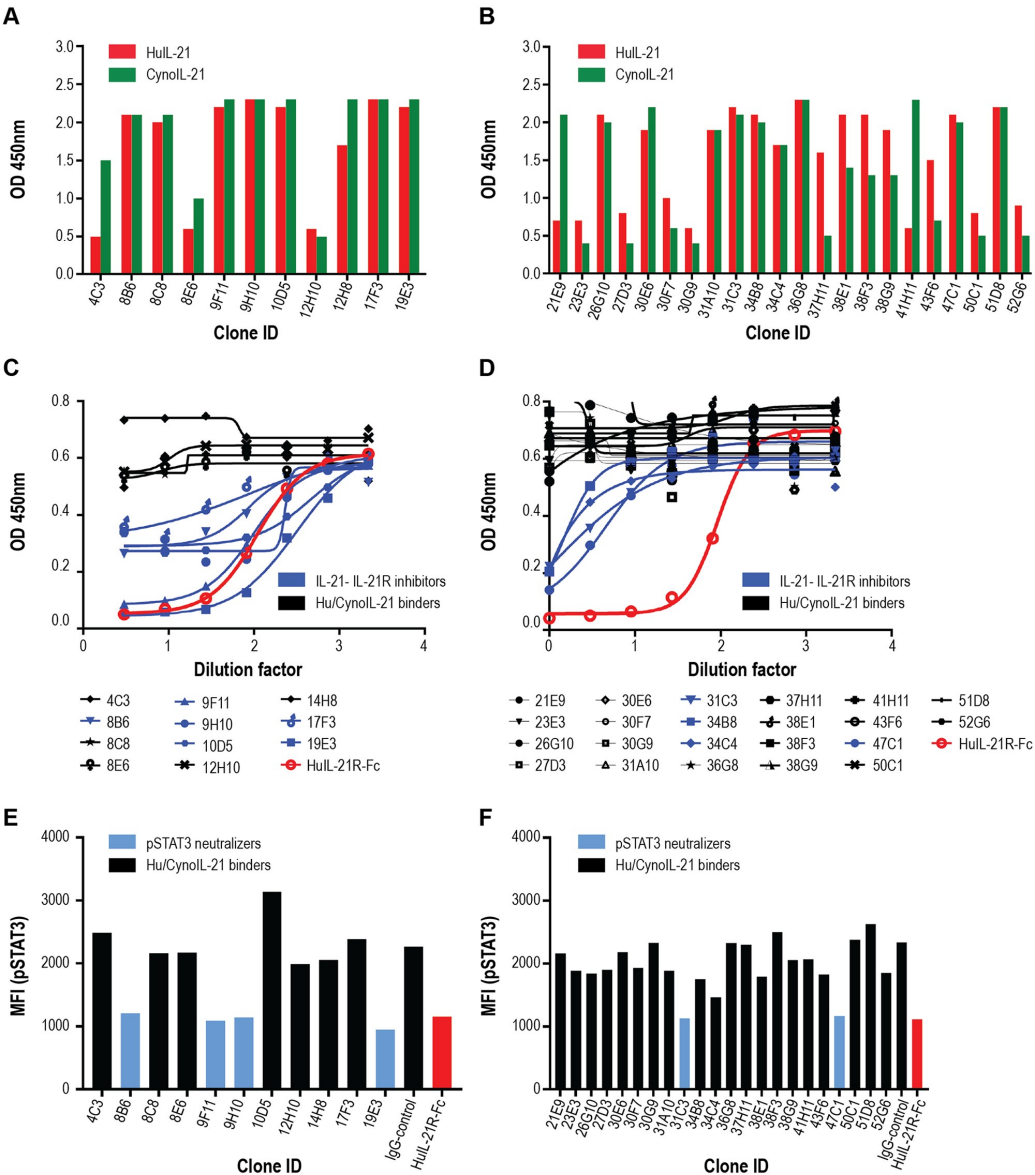
More efficient generation of neutralizing hybridomas against IL-21 from the alternating-antigen approach. (A, C, and E) hybridomas isolated from the alternating antigen approach. (B, D and F) hybridomas isolated from the single antigen approach. (A, B) Binding ELISA results of human and cyno IL-21 cross-reactive hybridoma supernatants. (C, D) Cross-reactive hybridoma supernatants were tested for their ability to inhibit the human IL-21/IL-21R-Fc interaction in a competition ELISA. Numbers on the x-axes are the dilution folds in log scale. The red lines represent the inhibitory activity displayed by IL-21R-Fc and are included for comparison. OD = optical density. (E, F) All cross reactive hybridoma supernatants were also tested for their ability to inhibit IL-21-induced phosphorylation of STAT3 and were assessed by flow cytometry. The red bars represent the neutralizing activity exhibited by soluble IL-21R-Fc blocking the IL-21 interaction and are included for comparison. Light blue bars denote anti-IL-21 clones which neutralize STAT3 phosphorylation.

Two mice from each group with serum neutralizing antibody titers of a thousand were selected for hybridoma generation. A total of 11 human and cyno IL-21 cross-reactive hybridomas were isolated from the dual-antigen group, and 22 species cross reactive hybridomas were identified from the single-antigen group (Fig 2A and 2B, respectively).

Ultimately antibodies that block IL-21 from binding to the receptor are sought, therefore supernatant from the individual hybridomas were screened for inhibition of human IL-21/IL-21R interactions (Fig 2C and 2D). Strikingly, more than 55% (6 of 11) of hybridomas from the dual-antigen group achieved significant (>30%) inhibition of human IL-21/IL-21R interactions as shown in blue. Notably, supernatant from two of these hybridomas, 9F11 and 19E3, achieved complete inhibition comparable to huIL-21R-Fc used as a control (Fig 2C). In mice immunized with human IL-21 alone, less than 20% (4 of 22) of hybridomas achieved significant inhibition (>30%), and none achieved complete inhibition (Fig 2D, blue).

The IL-21R is known to activate several signaling cascades, including molecules in the JAK/STAT pathway. Previous studies have demonstrated that activation of STAT3 is particularly critical for driving IL-21–mediated effects in human B cells [1]. To determine if the hybridomas block IL-21R signaling, all 33 supernatants were also tested for inhibition in the IL-21 induced STAT3 phosphorylation (pSTAT3) assay (Fig 2E and 2F). Of the six IL-21/IL-21R inhibitory hybridomas isolated from the dual antigen protocol shown in Fig 2C, four were potent neutralizers of the IL-21R signaling cascade (Fig 2E). Two (47C1 and 31C3) out of four weak IL-21/IL-21R inhibitors isolated from the single antigen group neutralized pSTAT3 activity comparable to the positive control (Fig 2F), however clone 31C3 was unstable and therefore not carried forward. From the original 33 hybridoma binders, the five remaining antibodies that inhibited STAT3 up-regulation were sub-cloned, expressed and purified for further characterization.

### Characterization of lead hybridoma clones

**Fig 3:**
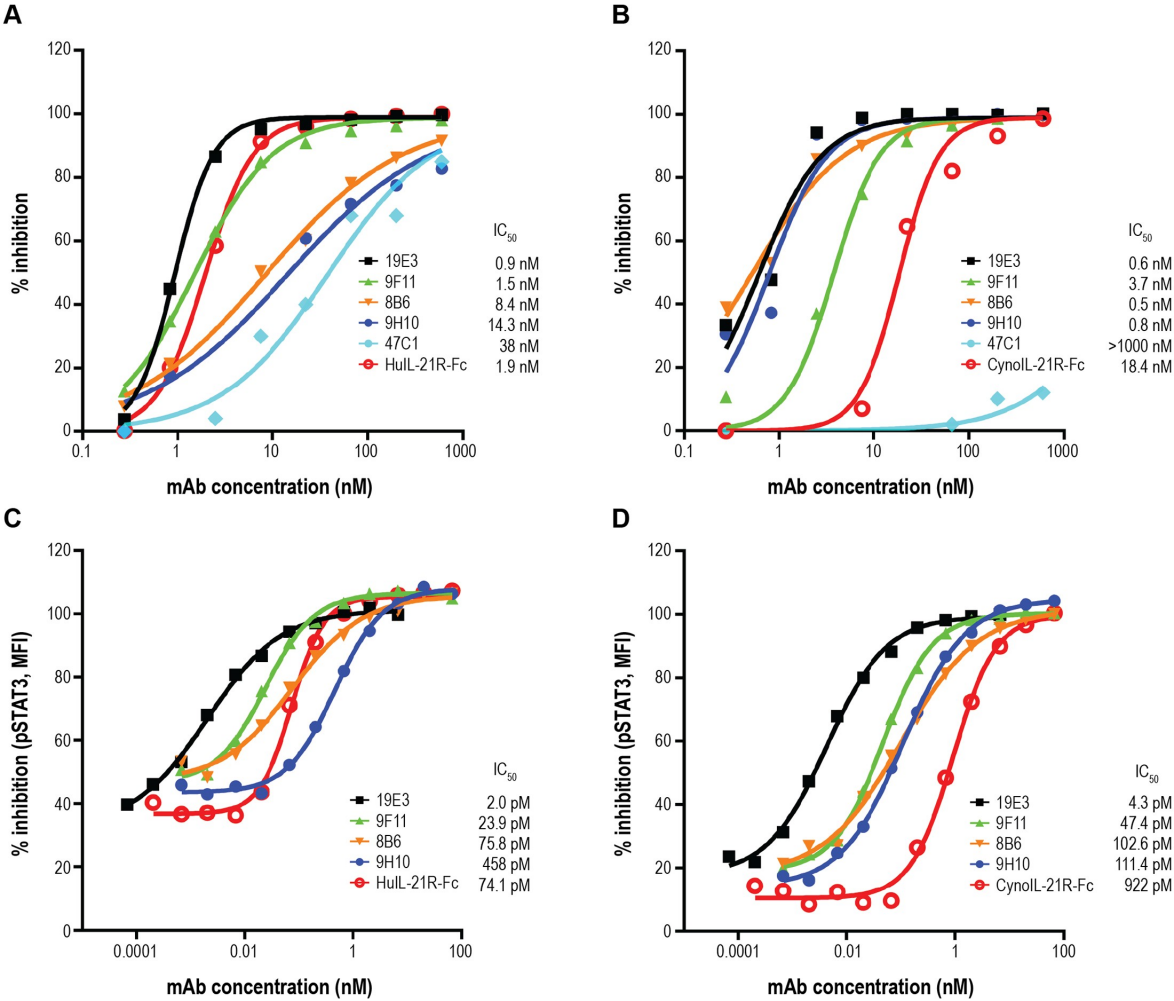
Lead hybridoma clones blocked receptor engagement and inhibited signaling through IL-21R. The 5 most potent inhibitory hybridoma clones were purified and re-tested in the inhibition assay against (A) human IL-21/IL-21R-Fc and (B) cyno IL-21/IL-21R-Fc interactions. Human PBMCs were stimulated for 15 min with (C) human or (D) cyno IL-21, and phosphorylation of STAT3 was quantified by flow cytometry. Graphs represent inhibition of IL-21–induced STAT3 phosphorylation by the indicated hybridoma clones. Data are representative of 3 donors tested. Percent inhibition was calculated as: 100*(1-(pSTAT3 MFI of stimulated+MEDI7169—pSTAT3 MFI of unstimulated)/(pSTAT3 MFI of stimulated—pSTAT3 MFI from unstimulated)).

Four clones (8B6, 9F11, 9H10 and 19E3) out of the 11 hybridomas that were isolated from mice immunized by the alternating antigen protocol blocked both human and cyno IL-21/IL-21R interactions in a competition ELISA and exhibited potent cross-species inhibitory activity (Fig 3A and 3B, respectively). Interestingly, these studies also revealed that 47C1, the only clone out of 22 hybridomas derived from the single antigen protocol, did not possess inhibitory activity against cyno IL-21 (Fig 3B, light blue), despite its strong binding activity for both human and cyno IL-21 by ELISA (Fig 2B). With robust cross-species inhibition of IL-21/IL-21R interactions, the four most potent antibodies (8B6, 9F11, 9H10 and 19E3) derived from the dual antigen protocol were evaluated in additional functional assays.

#### Blockage of IL-21–induced STAT3 phosphorylation by anti–IL-21 hybridoma clones

The ability of the lead hybridoma clones 8B6, 9F11, 9H10 and 19E3 to inhibit IL-21 induced phosphorylation of STAT3 was investigated using human peripheral blood mononuclear cells (PBMCs) in response to human and cyno IL-21 (Fig 3C and 3D, respectively). All 4 hybridoma clones tested completely inhibited phosphorylation of STAT3 in a dose-dependent manner, in most cases more potently than did the recombinant IL-21R-Fc. Hybridoma clone 19E3 was the most potent antibody, having 50% inhibitory concentrations (IC_50_) of 2 pM against human IL-21 and 4 pM against cyno IL-21 in this assay.

#### Epitope binning and binding kinetics of isolated chimeric hybridoma clones

**Fig 4:**
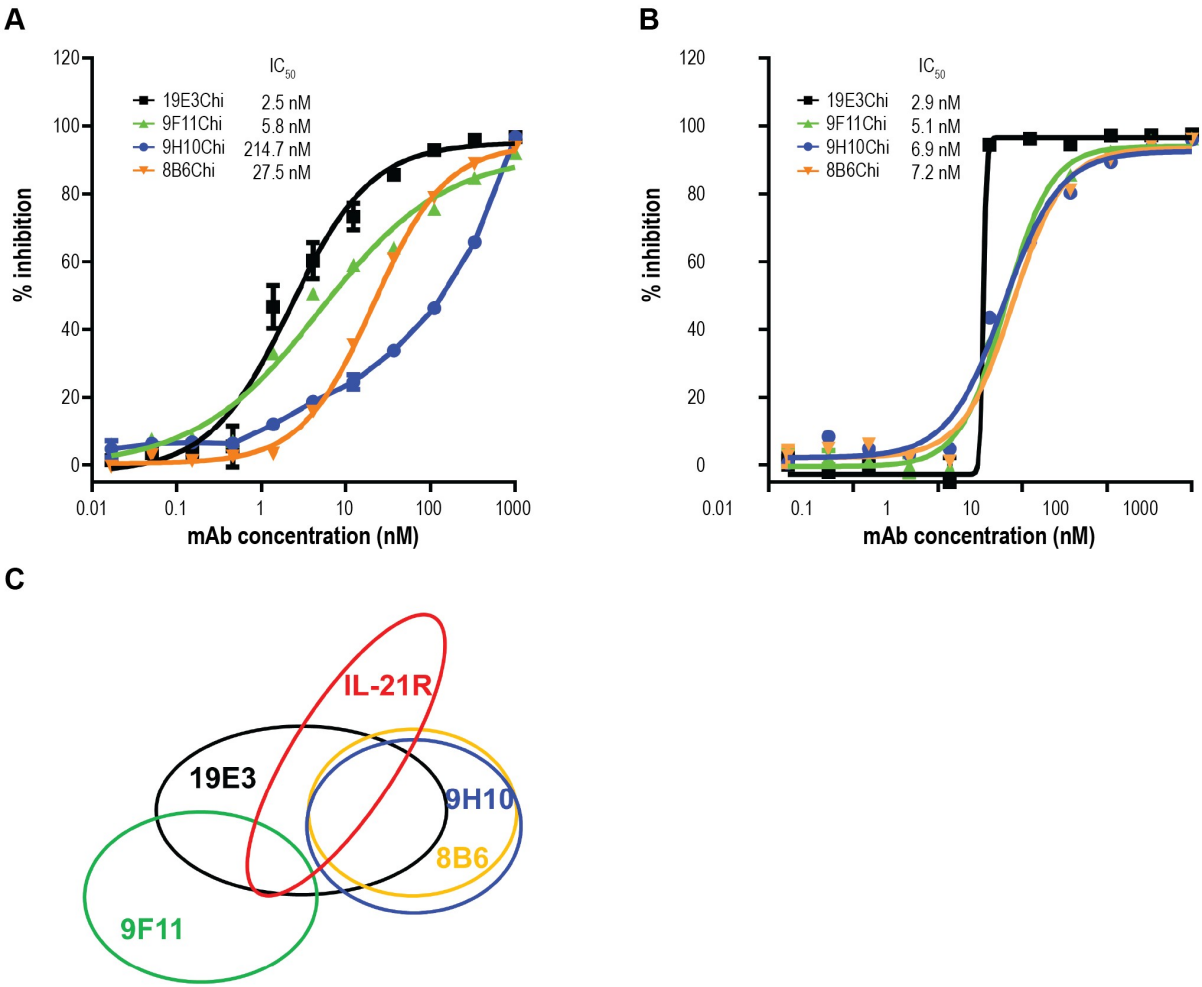
Activity, epitope binning, and affinity of chimeric hybridoma clones. The 4 most potent inhibitory hybridoma clones were molecularly cloned and expressed as murine/human chimeric IgGs 8B6Chi, 9F11Chi, 9H10Chi, and 19E3Chi, and were characterized for inhibition against (A) human or (B) cyno IL-21/IL-21R-Fc interactions, and (C) epitope bin distribution of the 4 chimeric antibodies.

To further characterize the 4 lead clones, 8B6, 9F11, 9H10, and 19E3 were constructed as chimeric IgGs containing murine antigen-binding fragment (Fab) and human Fc. As shown in Fig 4A and 4B, the ability of these chimeric IgGs to inhibit IL-21/IL-21R interactions was confirmed in the ELISA competition assay. Interestingly, these 4 chimeric IgGs demonstrated differentiating IC_50_s against human IL-21/IL-21R, but similar IC_50_s against cyno IL-21/IL-21R (Fig 4A and 4B, respectively). The epitopes of the four chimeric hybridoma clones were characterized by competition binding assay, using Bio-layer interferometry (Octet, Pall ForteBio, Fremont, CA). In this study, two mAbs that can simultaneously bind IL-21 belong to different epitope bins, while mAbs that compete for binding to IL-21 share the same or similar, overlapping epitopes. The data showed that 19E3 bound an overlapping epitope with the other three antibodies. While 8B6 and 9H10 shared a similar epitope with each other, 9F11 bound an epitope distinct from them (Fig 4C). All 4 clones bound overlapping epitopes with IL-21R-Fc, which is consistent with their ability to block the activity of IL-21/IL-21R interactions. The binning data revealed that the alternating-antigen immunization effectively generated IL-21–neutralizing antibodies with overlapping but not identical epitopes, which could be advantageous when developing best-in-class molecules.

The kinetics of binding and affinity of the 4 chimeric clones to human IL-21 were determined using a kinetic exclusion assay (KinExA; Sapidyne Instruments, Boise, ID). The association (K_on_) and dissociation (K_off_) rate constants and derived affinity (K_D_) are shown in Table 1.

**Table 1:** Affinity of hybridoma clones to human IL-21.

IgG	K_D_ (pM)	K_on_ (1/Ms)	K_off_ (1/s)
**19E3**	0.24	1.52 x 10^7^	3.72 x 10^−6^
**9F11**	10	8.30 x 10^7^	829 x 10^−6^
**9H10**	108	5.4 x 10^6^	583 x 10^−6^
**8B6**	7.6	2.54 x 10^6^	19.3 x 10^−6^

Affinity (K_D_), association/dissociation rate constant (K_on_/K_off_) was

determined by a kinetic exclusion assay (KinExA) as described in Methods.

Among the four lead clones, 19E3 had the highest affinity to recombinant human IL-21 of 243 fM. Because of its high affinity for the target and its potent suppression of IL-21 signaling, clone 19E3 was further evaluated in a full panel of biological assays.

### 19E3 inhibition of PC differentiation induced by exogenous IL-21

**Fig 5:**
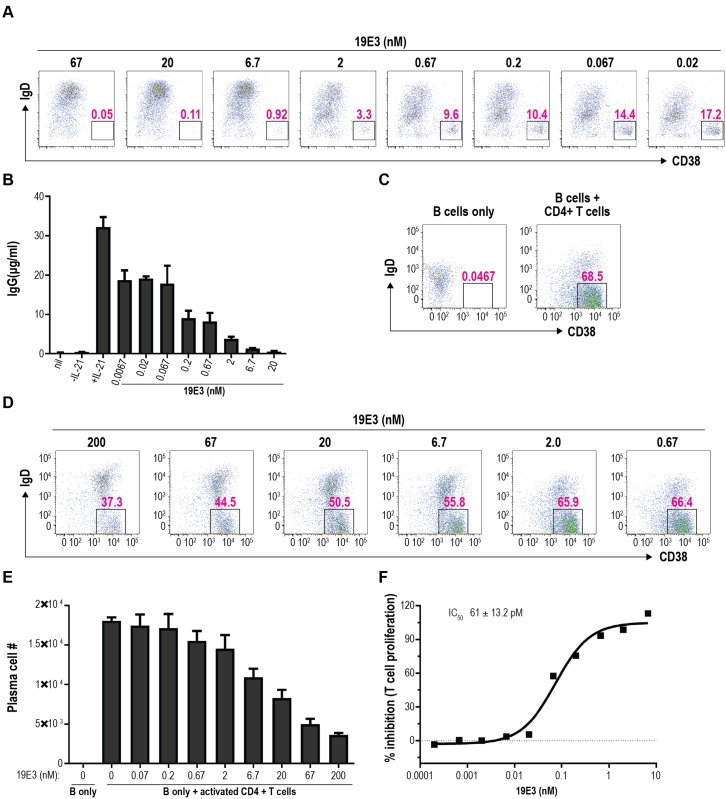
19E3 inhibition of PC differentiation mediated by recombinant and endogenous IL-21. (A, B) B cells were isolated from human peripheral blood and stimulated with or without recombinant IL-21 in combination with anti-CD40 and anti-IgM. 19E3 was included at the indicated concentrations. (A) PCs were quantified on day 7 by flow cytometry as IgD^–^ CD38^hi^ cells (percent of PCs in well indicated). All samples were run in duplicate. (B) Supernatants were collected on day 7 and IgG levels were quantified by ELISA. The experiment was performed with 5 unique donors in 3 separate experiments. Results from a representative donor are shown. (C, D and E) Human B cells were co-cultured with anti-CD3/anti-CD28–activated CD4^+^ T cells in the presence of graded doses of 19E3. After 7 days of co-culture, PCs, defined as CD19^+^ IgD^-^ CD38^hi^ cells, were quantified by flow cytometry. Percent (C, D) and number (E) of PCs for each condition are shown. The experiment was performed with 3 unique donors in 2 separate experiments. Results from 1 representative donor are shown. (F) Human T cells were stimulated with recombinant human IL-21 in combination with anti-CD3. 19E3 was added at the indicated concentrations and T-cell expansion was quantified on day 4. All conditions were run in duplicate. The experiment was performed with 2 unique donors. Results from a representative donor are shown.

Previously we have shown that IL-21 has a profound impact on B-cell survival, activation, and proliferation, as well as on the differentiation of B cells into immunoglobulin-secreting PCs [6, 34, 35]. Therefore, we next determined the ability of 19E3 to inhibit IL-21–driven PC differentiation. Stimulation of human B cells with human IL-21 in combination with anti-CD40 and anti-IgM F(abʹ)_2_ resulted in the generation of an IgD^–^ CD38^hi^ PC population. Clone 19E3 completely inhibited IL-21–induced PC differentiation in a dose-dependent manner (Fig 5A).

In addition, stimulation of B cells with IL-21, anti-CD40, and anti-IgM in the presence of a negative control antibody resulted in IgG production by the differentiated PC in the range of 30 to 40 μg/mL by day 6 (Fig 5B). Notably, clone 19E3 reduced IgG secretion in a dose-dependent manner, and completely suppressed IgG production at the highest concentration tested (Fig 5B). Cumulatively, these data suggest that 19E3 significantly inhibited PC differentiation and ensuing IgG secretion induced by exogenous human IL-21.

#### 19E3 inhibition of PC differentiation induced by activated CD4^+^ T cells

Activation of B cells *in vivo* can be driven by interactions with activated T cells, which express costimulatory molecules such as CD40L and produce B-cell tropic cytokines such as IL-21. Previously we have shown that IL-21 is required for efficient PC differentiation *in vitro* in response to activated T cells [35]. Consequently, 19E3 was tested for its ability to block PC differentiation induced by activated CD4^+^ T cells in a co-culture assay. Co-culture of B cells with anti-CD3/CD28–activated CD4^+^ T cells resulted in the emergence of an IgD^–^ CD38^hi^ PC population; 68.5% of the CD19^+^ cells expressed this PC phenotype by day 7 of culture (Fig 5C). Addition of 19E3 inhibited PC differentiation in a dose-dependent manner (Fig 5D), achieving a maximum inhibition of 80% in this assay (Fig 5E). These data suggest that 19E3 can largely block PC differentiation induced by *de novo* IL-21 production from activated T cells.

#### 19E3 blockade of IL-21–induced T-cell expansion

IL-21 has been shown to support CD4^+^ T-cell proliferation under suboptimal priming conditions [36]. Human naïve CD4^+^ T cells were purified and stimulated in vitro with anti-CD3 and IL-21 in the presence or absence of 19E3. Proliferation was assessed by measuring the accumulation of ATP after 4 days in culture. 19E3 blunted IL-21–induced proliferation (average IC_50_ = 61± 13pM for 2 donors) in a dose-dependent manner. These data show that clone 19E3 inhibits IL-21–driven proliferation of human naïve CD4^+^ T cells (Fig 5F).

### Humanization and engineering of 19E3

**Fig 6:**
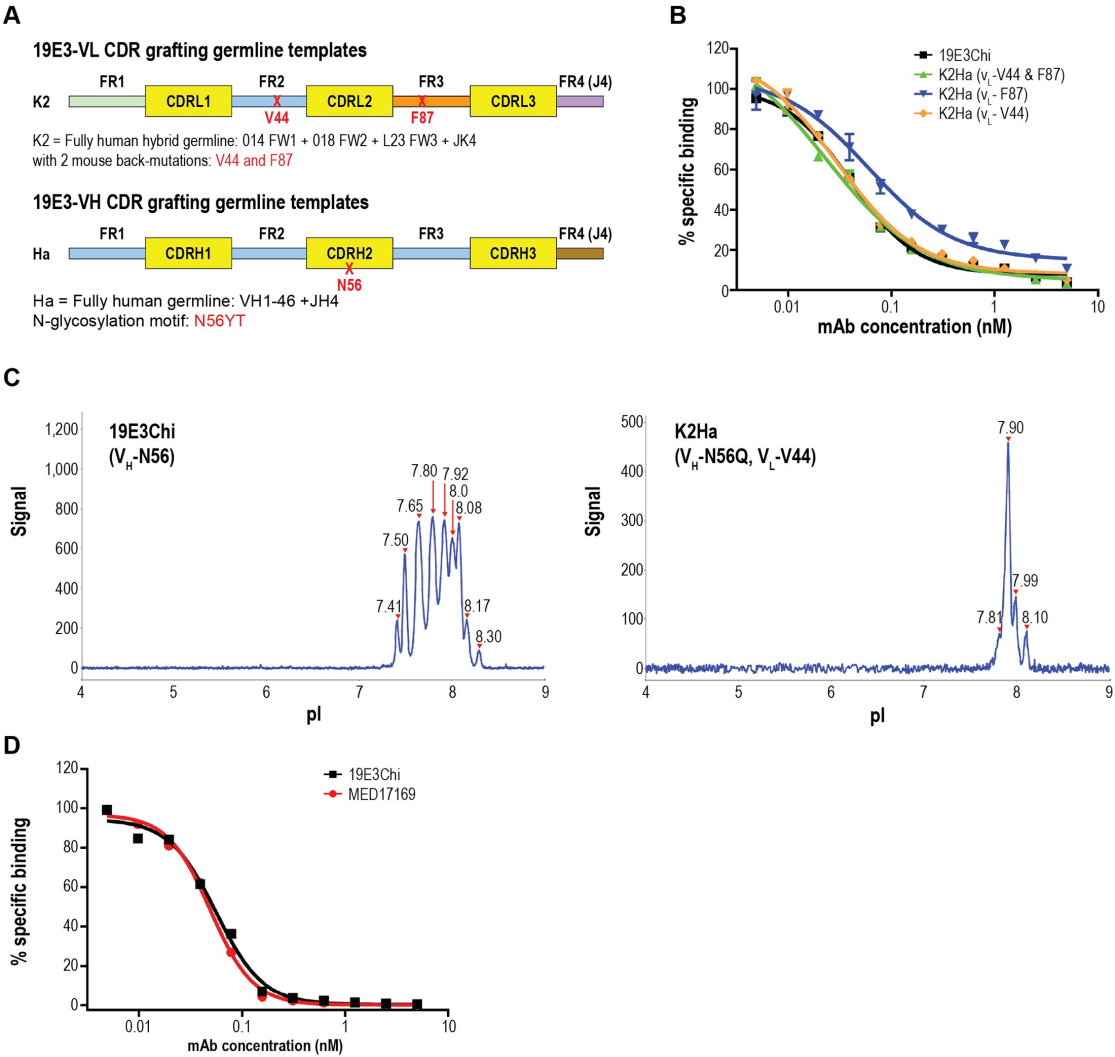
Humanization and engineering of hybridoma clone 19E3. (A) Humanization design strategy: K2 light-chain acceptor framework template combined different human germline FRs and encoded 2 back-mutated murine residues (V44 and F87) illustrated by the 2-red x’s. Ha heavy-chain acceptor framework template designed from one human germline. An N-glycosylation site in the V_H_-CDR2 is shown. FRs from different germlines are highlighted with contrasting color blocks. (B) Humanized clones screened by HTRF epitope competition assay: Unlabeled humanized variants competed with europium cryptate–labeled chimeric mAb 19E3 for binding to biotinylated IL-21. Unlabeled 19E3Chi was used as control to demonstrate 100% binding. K2Ha (V_L_-V44 and F87), encoding two murine residues, and K2Ha (V_L_-V44) encoding only one murine residue both exhibited binding activity comparable to 19E3Chi. Encoding one murine residue K2Ha (V_L_-F87) resulted in decreased binding activity. (C) Mitigation of N-glycosylation site: NanoPro cIEF electropherogram of 19E3Chi with V_H_-N56 glycosylation site and K2Ha (V_L_-V44) with the glycosylation site removed (V_H_-N56Q). Multiple peaks indicate more charge heterogeneity. Charge isoforms were aligned through calibration of the internal fluorescence pI markers. (D) HTRF epitope competition assay. The final humanized lead mAb, MEDI7169, demonstrated the same binding activity as 19E3Chi.

To minimize the risk of immunogenicity, clone 19E3 was humanized by grafting its murine CDRs into the human germline sequences [37]. Practicing this method often results in reduced affinity for the target antigen and restoring the affinity through additional engineering is necessary [31]. Here, to maintain the sub-picomolar affinity of 19E3, a unique donor germline selection method was employed. Individual FRs from multiple human germlines were selected to design an optimal hybrid germline acceptor template, rather than using one germline or a consensus sequence for the entire light- (V_L_) or heavy- (V_H_) chain variable region. This concept was adopted from the FR shuffling method [38, 39] but differed by its empirical approach and rational design. When selecting human germline FRs to achieve the highest homology possible with their murine counterparts, several criteria were carefully considered, such as matching critical residues in the Vernier zone, canonical residues, and V_H_/V_L_ interface residues, [40–42], and avoiding potential immunogenicity by considering germline frequency and the percent homology to the donor framework. As illustrated in Fig 6A, three human germline FRs, O14 (FR1), O18 (FR2), and L23 (FR3), were selected and combined with JK1 (FR4) to serve as the V_L_ acceptor template. The three best human V_H_ FRs, each of which were individually matched using the above criteria to their corresponding murine FRs, came from the same human germline V_H_1-46. Combined with JH4, this was identified as the V_H_ acceptor template named Ha. Although the FR (excluding CDRs) homology between the best-matched human templates and 19E3 was about 80% for V_L_ (with 16 mismatched FR residues) and 73.5% for V_H_ (with 23 mismatched FR residues), the critical murine FR residues deemed to be essential for structural chain conformations, light and heavy chain interactions, or for support of the CDR loops differed by only 2 amino acids (aa) in the V_L_ and 4aa in the V_H_ human templates. These two murine V_H_/V_L_ interface residues, V44 and F87, were selectively back-mutated onto the V_L_ human template and named K2 [43]. A panel of humanized IgG variants were generated and screened by homogeneous time-resolved fluorescence (HTRF) epitope competition assay [44] which is based on the displacement of the parental 19E3 binding to IL-21 by the unlabeled humanized clones. The binding activity and affinity of the humanized clones can be easily ranked against the parental 19E3 in this assay. Pairing the humanized V_L_ chain K2 (V_L_-V44, V_L_-F87) which retains two murine FR residues built in the initial V_L_ template, with the fully humanized V_H_ variant Ha resulted in a humanized antibody (K2Ha) with retained IL-21 binding comparable to the murine parent mAb (Fig 6B, green). Strikingly, the V_L_ framework was further engineered to reduce the murine back mutations, encoding only the single murine residue of V44 while maintaining the sub-picomolar binding affinity of the parental antibody 19E3 (Fig 6B, blue).

A high-risk, N-linked glycosylation motif (NYT) was identified in the V_H_-CDR2 of 19E3 at position N56. Heterogeneity caused by glycosylation in the variable region, in addition to Fc glycosylation, should be avoided for product quality control in manufacturing [45]. NanoPro (ProteinSimple, San Jose, CA) is an imaged capillary isoelectric focusing (cIEF) immunoassay that detects separated proteins by their isoelectric points (pIs) and has been used for charge heterogeneity analysis of mAb products [46] cIEF analysis showed the electropherograms of 19E3Chi (V_H_-N56) with multiple peaks (Fig 6C, left), indicative of the charge heterogeneity caused by the N-linked glycosylation. However, replacing N with Q to remove the same NYT motif from the humanized clone K2Ha (V_L_-V44) resulted in much cleaner electropherograms with fewer peaks (Fig 6C, right), a profile typical of an antibody with N-linked glycosylation at residue N297 in the Fc region. These data suggest that the observed charge heterogeneity was associated with N-glycosylation in the Fab region. The final humanized and engineered antibody, K2Ha (V_H_-N56Q, V_L_-V44), retained only 1 murine FR residue in the light chain, eliminated the N-glycosylation site in the V_H_-CDR2, and demonstrated IL-21 binding activity comparable to that of chimeric19E3 (Fig 6D). This clone was designated as MEDI7169. Affinity constants measured by KinExA and reported in Table 2 indicated that MEDI7169 retained a sub-picomolar affinity to human and cyno IL-21, comparable to the parental antibody 19E3 (Table 1).

**Table 2:** Affinity of MEDI7169 to human and cyno-IL-21.

	Affinity (human IL-21)			Affinity (cyno IL-21)		
	K_D_ (fM)	K_on_ (1/Ms)	K_off_ (1/s)	K_D_ (fM)	K_on_ (1/Ms)	K_off_ (1/s)
**MEDI7169**	515	1.72 x 10^7^	8.85 x 10^−6^	352^a^	NA^b^	NA^b^

Affinity (K_D_), association/dissociation rate constant (K_on_/K_off_) was determined by a kinetic exclusion assay (KinExA) as described in Methods.

^a^ Determined from equilibrium binding,

^b^NA = not applicable

### MEDI7169 inhibition of IL-21 activity

**Fig 7:**
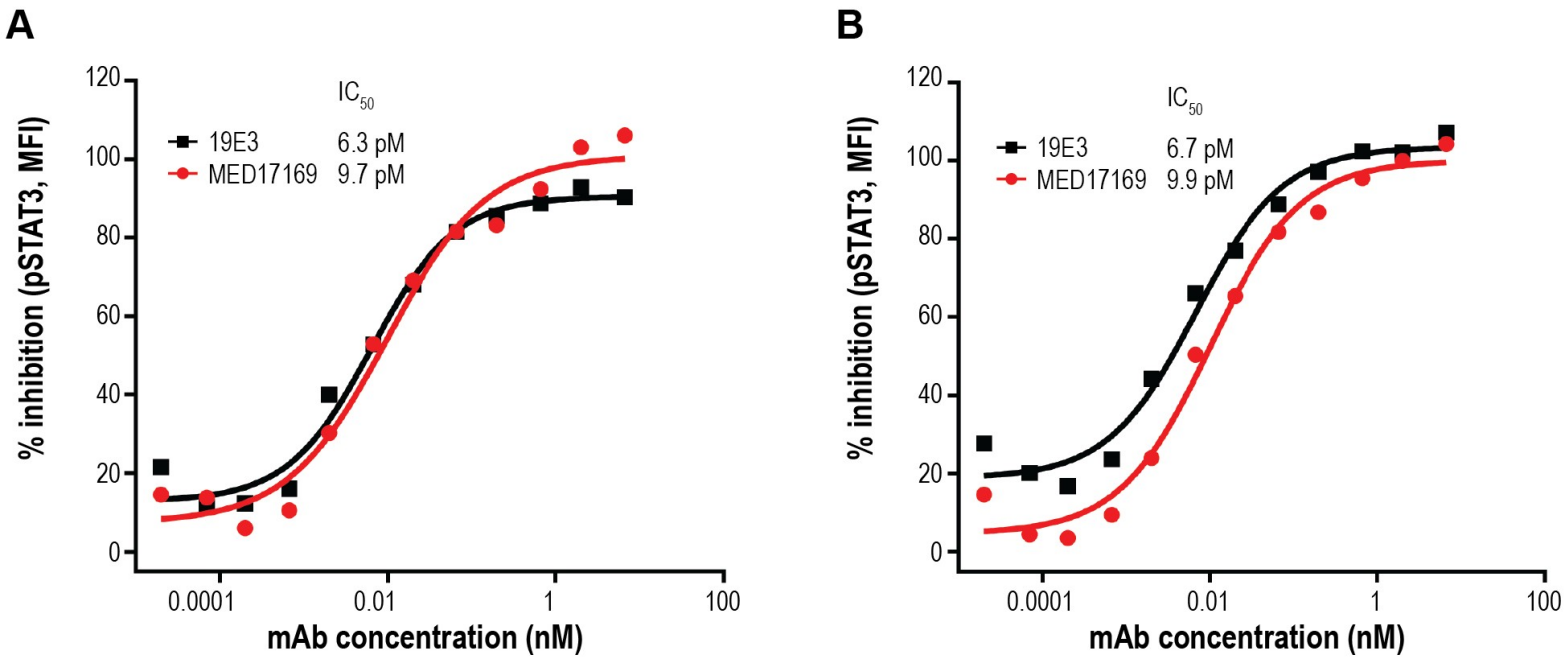
MEDI7169 inhibition of IL-21–induced STAT3 phosphorylation. Human PBMCs were stimulated with recombinant (A) human or (B) cyno IL-21 in the presence of MEDI7169 as indicated. Phosphorylation of STAT3 was assessed by flow cytometry. Percent inhibition of pSTAT3 induction is shown. All conditions were run in duplicate; 1 of 5 representative donors from 4 independent experiments is shown.

To confirm the biological activity of the humanized clone MEDI7169, we tested its inhibition of IL-21/IL-21R interactions by ELISA and IL-21–induced phosphorylation of STAT3 using human PBMCs in response to human and cyno IL-21. MEDI7169 demonstrated IC_50_ values comparable to those of 19E3 for human (3.7 vs 2.5 nM, respectively) and cyno (2.1 vs 2.9 nM, respectively) IL-21/IL-21R interactions (Table 3). MEDI7169 completely inhibited pSTAT3 upregulation induced by both human and cyno IL-21. IC_50_s of MEDI7169 in this assay were 9.71 pM for human and 9.99 pM for cyno, comparable to the potency of the parental antibody 19E3 (Fig 7A and 7B, respectively).

**Table 3:** MEDI7169 inhibition of human or cyno IL-21/IL-21R-Fc interactions.

	IC_50 (nM)_	
	Human IL-21/IL-21R	Cyno IL-21/IL-21R
**19E3**	2.5 ± 0.3	2.9 ± 1.1
**MEDI7169**	3.7 ± 1.6	2.3 ± 0.3

Inhibition activity and IC_50_ values were determined in the ELISA-based competition assay as described in Figs 2, 3 and 4.

### Specificity of MEDI7169 to IL-21

**Fig 8:**
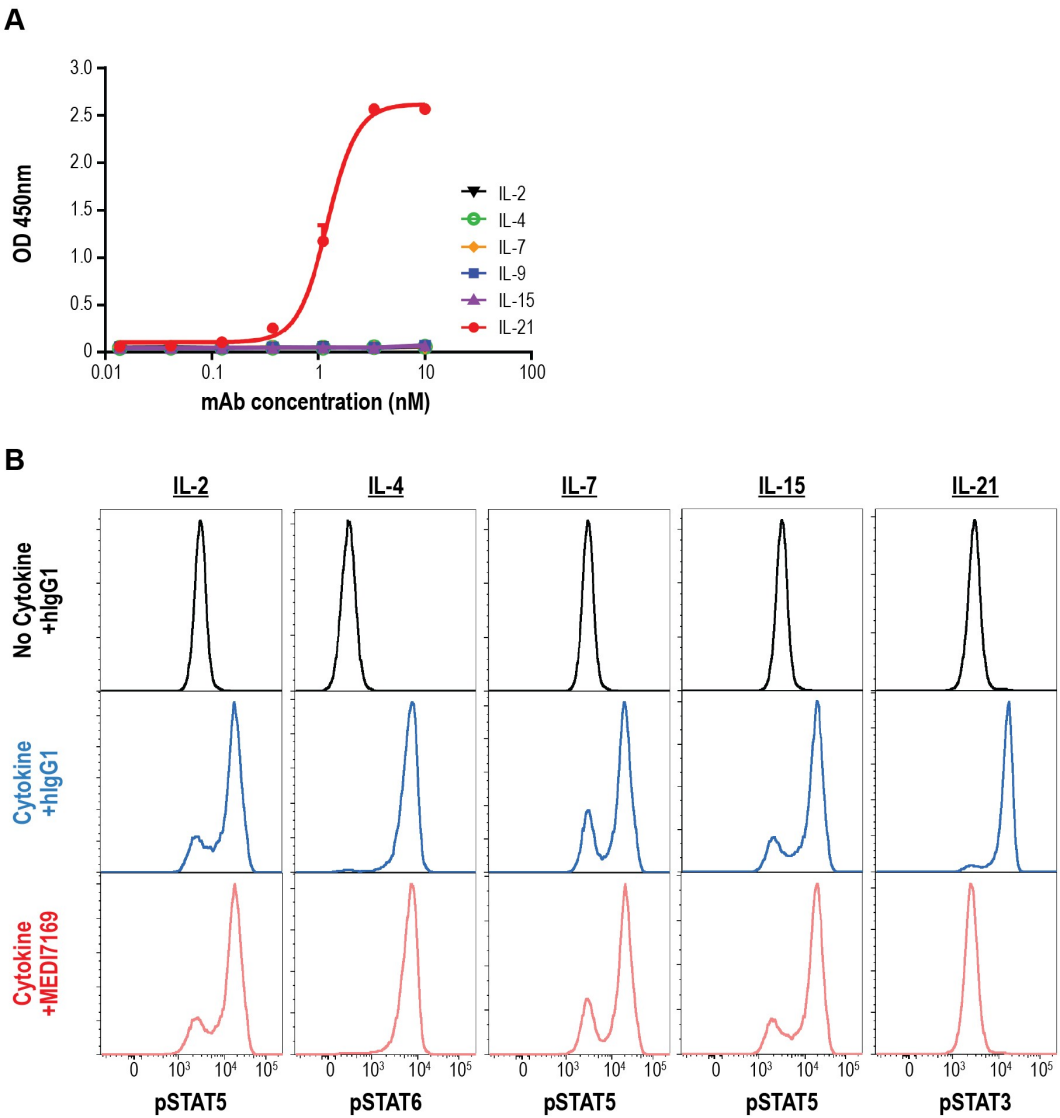
MEDI7169 binding specificity and neutralization of IL-21. (A) The binding specificity of MEDI7169 to common γc cytokines (IL-2, IL-4, IL-7, IL-9, IL-15, and IL-21) was evaluated by ELISA. Samples were tested in duplicate wells. Values are expressed as mean ± standard deviation. (B) Human PBMCs were stimulated with recombinant human IL-2, IL-4, IL7, IL-15, or IL-21 in the presence of MEDI7169 or human IgG1 control antibody. Cytokine-induced phosphorylation of STAT molecules appropriate for each cytokine was assessed by flow cytometry. Induction of STAT phosphorylation signalling in the presence of MEDI7169 or human IgG1 is shown for each cytokine vs no cytokine control. All conditions were run in duplicate. Results from 1 of 2 representative donors are shown. OD = optical density.

Along with IL-21, other γ_c_ cytokines including IL-2, IL-4, IL-7, IL-9, and IL-15, signal through STAT proteins [47, 48] The binding specificity of MEDI7169 to IL-21 versus other γ_c_ cytokines was evaluated in an ELISA binding assay. MEDI7169 exhibited specific binding only to IL-21 (Fig 8A). Furthermore, the specificity of MEDI7169 was tested for its ability to influence IL-2, IL-4, IL-7, IL-15, or IL-21 downstream signaling events by assessing the phosphorylation of STAT molecules appropriate for each cytokine. MEDI7169 had no effect on STAT phosphorylation induced by IL-2, IL-4, IL-7, or IL-15 cytokines but completely inhibited IL-21–induced pSTAT3 upregulation (Fig 8B). These data show that MEDI7169 acts specifically to neutralize the IL-21 pathway.

## Discussion

Hybridoma continues to be one of the major therapeutic antibody discovery platforms. More than 4 decades after its inception, the platform has not only evolved but continues to generate attractive leads. The advent of automation has simplified the screening process and has drastically reduced timelines. Genetically engineered mice have become robust platforms for the generation of diverse repertoires of affinity-matured, fully human antibodies. Despite all these advances, the hybridoma platform still has some limitations, as immunizations are carried out *in vivo* and do not allow for complete control over the entire process. Other antibody discovery methods offer more control; for example, phage panning can be performed with multiple antigens in a sequential manner so that antibodies targeting conserved epitopes can be enriched, and in the process, antibodies targeting epitopes not shared by all the antigens can be eliminated. To translate the same concept into animal immunizations is highly challenging from a practical standpoint. Because B-cell clones targeting either conserved or non-conserved epitopes cannot be removed during immunization, the outcome of multiple-antigen exposure could be multi-pronged: All B cells could evolve in parallel, delivering antibodies with the greatest diversity to the immunodominant epitopes that are conserved between the species, but without any bias for a specific epitope (antibodies against the entire antigen, isolating binders that may be functional). Alternatively, B cells specific for unique or functional epitopes could become enriched towards the newly introduced antigenic regions and are amplified due to persistent alternating-antigen stimulation.

Anti–IL-21 antibody generation presents an outstanding opportunity to address these questions, which are of fundamental importance to immunology and the hybridoma platform. Given the low homology (61–65%) of human and rodent IL-21 it was unlikely to isolate rodent cross-reactive antibodies. Therefore, we selected cyno IL-21 as the non-human primate species relevant for preclinical evaluation. Alternating human & cyno IL-21 presented an ideal opportunity to test our hypothesis while delivering an antibody with therapeutic potential. Despite the high degree of homology shared between human and cyno IL-21, they did not recognize either IL-21R equally. In fact, human IL-21 bound cyno IL-21R better than its cognate ligand (S1 Fig), which could be attributed to the 1 amino acid different between species within the conserved receptor binding region of IL-21. We therefore wanted to investigate whether alternating the same antigen from two species during immunization could direct the immune response toward this conserved, IL-21R–binding epitope on both human and cyno IL-21. We observed significant differences in serum responses in the two groups of mice; those receiving alternating-antigen immunization displayed potent neutralizing activity, whereas mice receiving only 1 antigen had quantitatively and qualitatively less neutralizing activity. Though both platforms delivered human and cyno IL-21 cross-reactive binders, potent neutralizing hybridomas were only isolated from the group receiving two antigens, suggesting that the immune system has the capacity to distinguish between and hone in on the conserved epitopes existing on similar yet distinct antigens. Antibodies derived from the single antigen platform recognized the cyno IL-21 antigen, but without selective pressure towards functional epitopes, failed to block the ligand/receptor interaction. We hypothesize that with this alternating immunization strategy antibodies were first raised in response to human IL-21 in its entirety, recognizing both human and cyno IL21 due to their high degree of homology and consequently conserved epitopes. After the introduction of new antigenic epitopes present on cyno IL-21, antibodies which recognized regions containing the six differing residues of the cyno IL-21 were enriched. The additional boost with human IL-21 directed the immune response to hone in on antibodies that recognize both species in these unique positions, giving rise to not only cross-reactive but most importantly cross neutralizing antibodies. Therefore, alternating antigen exposure enriched for antibodies that recognized unique epitopes encompassed within conserved functional domains. Of all the high affinity binders that were isolated from both methods only one recognized but did not neutralize muIL-21. As expected, the more distant the sequence homology the more challenging it becomes to dial in species cross reactivity. This concept is further supported by the results of two recent reports [26, 27] where sequential immunization with various mutant versions of the antigen against HIV infection, or mixing multiple toxin antigens together during immunization both succeeded in inducing broadly neutralizing antibodies. Like our study, both approaches capitalized on the immune response to different antigenic epitopes to converge on important functional domains. Our new immunization strategy, along with these methods, should be exploited to further improve therapeutic mAb discovery and vaccine immunogen designs.

One of the strengths of the hybridoma platform is in the *in vivo* maturation of the antibodies isolated from the mice. Indeed, the four murine mAbs profiled in this study demonstrated binding KDs in the picomolar range, yet still had the potential to elicit an anti-drug antibody (ADA) response if administered to humans. Given the ultra-high femtomolar affinity of the lead hybridoma 19E3, all efforts were made to maintain the binding, specificity, and neutralization potency in the humanized version of this monoclonal. Instead of choosing a single human germline template for the entire V_L_ when applying the CDR grafting method for the humanization of 19E3, we unconventionally used 3 different human germlines as the acceptor framework. Key to this design strategy is considering the critical residues within the murine FRs when selecting the human germlines with the highest homology as the acceptor framework (Vernier zone, canonical class, and V_H_/V_L_ interface) that could impact antigen binding. In this case, we identified only 2 murine V_H_/V_L_ interface residues that differed from their human counterparts and mutated them back onto the V_L_ human template, while changing the other 16 mismatched FR residues between the murine and human V_L_ FRs to reduce immunogenicity. As a result, the hybrid framework sequence matched better to the parent mouse mAb than a single human germline at most of the residues deemed critical for maintaining the CDR loop conformation. Subsequently only 1 murine residue was retained in MED7169 V_L_ framework. A similar concept was reported in a different, empirical humanization method called Framework Shuffling, which involved building a comprehensive library of human germline FR genes, then utilizing the agnostic selective pressure of a high throughput screening assay to identify the most favorable human FRs [39]. Here, our rational design method, rarely reported as a direct application, utilized multiple germlines for CDR grafting. Our approach does not require any *a priori* structural knowledge of the parental antibody, which is often the case at this stage of antibody drug development. Incorporating the pre-identified critical murine FR residues into the human templates allowed for quick evaluation of the individual residues indirectly contributing to antigen binding. In this way, by designing just 2 to 3 human templates for each V_L_ and V_H_, we could identify humanized clones that retained the ultra-high affinity comparable to the parental antibody in a single round of screening. This method of antibody humanization is more efficient than conventional CDR grafting, which may involve several design cycles and an empirical endeavor to screen critical back-mutated parental residues.

N-linked glycosylation consensus sequences N-X-T/S (where X cannot be proline) exist in the variable region of about 20% of human IgGs [49]. N-glycosylation in the mAb variable region could have a positive, neutral, or negative effect on antigen binding [50, 51]. Numerous studies have reported the N-glycosylation heterogeneity and its potential impact on structure, function, pharmacokinetics, and pharmacodynamics of mAbs [52, 53]. N-glycosylation in the variable region in addition to Fc glycosylation results in protein heterogeneity which impedes product quality control. Controlling N-glycosylation during the manufacturing process is very costly for chemistry, manufacturing, and control development. In addition, glycan compositions in the variable region could potentially cause immunogenicity [52]. Therefore, we proactively mitigated the N-glycosylation motif NYT in the V_H_-CDR2 of humanized 19E3 without affecting its binding or biological function.

Most importantly, our results from a series of biological functional assays confirm that consequential to retaining sub-picomolar affinity, the humanized lead mAb MEDI7169 demonstrated high neutralization potency against IL-21, comparable to the parental 19E3. MEDI7169 retained only 1 murine residue in the V_L_ framework with N-glycosylation site removed, minimizing its risk of immunogenicity and increasing its developability and manufacturability potential.

## Conclusion

Here we describe a novel immunization platform and a unique humanization strategy. When applied, we efficiently isolated and effectively optimized a species cross-reactive, IL-21-specific, ultra-high affinity, neutralizing antibody MEDI7169. This molecule potently suppresses human lymphocyte responses in vitro, including plasma cell differentiation and antibody production, highlighting its potential utility in settings of autoimmunity where pathogenic autoantibodies contribute to disease progression, such as in SLE where MEDI7169 is currently being clinically evaluated in a phase 1b trial. Furthermore, the novel immunization and humanization principles reported here could be applied to other targets for a streamlined, drug discovery process where species cross-reactivity and functionality is desired.

## Materials and methods

### Data

All experiments described in this work are representative of at least two independent measurements.

### Reagents and cells

Recombinant human and cyno IL-21 and human and cyno IL-21R-Fc were generated at MedImmune (Gaithersburg, MD). Recombinant human and cyno IL-21 were biotinylated using the EZ-Link Sulfo-NHS-LC-Biotin labeling kit (catalog no. [cat#] 21327; Thermo Scientific, Waltham, MA). MEDI7169 is commercially available from Creative Biolabs. https://www.creativebiolabs.net/Anti-IL21-Antibody-75336.htm.

Human whole blood was collected after informed written consent from healthy volunteers recruited by the MedImmune Blood Donor program. Healthy control donors consisted of healthy MedImmune or AstraZeneca employees who were anonymously enrolled in the MedImmune Research Specimen Collection Program. The blood collection procedure was approved by the Chesapeake Institutional Review Board (protocol 2010–001). PBMCs were isolated from cell preparation tubes (cat# 362753; BD Biosciences, San Jose, CA) after centrifugation according to the manufacturer’s instructions. Total B cells and total CD4_+_ T cells were negatively selected using magnetic-activated cell separation technology (MACS; cat# 130-091-151 and 130-091-155; Miltenyi Biotec, Auburn, CA), which routinely yielded greater than 95% purity. Naive CD4^+^ T cells were negatively selected from PBMCs by using cell separation reagents (cat# 19155; StemCell, Cambridge, MA) according to the manufacturer’s instructions. Routine purity for naive CD4^+^ T-cell isolation is >97%.

### Biochemical screening assay setup

ELISA was used as the primary screening method for both mouse serum testing and identification of IL-21–specific human and cyno cross-reactive hits generated from the hybridoma campaign. For direct binding ELISA, human IL-21, cyno IL-21, BSA, and histidine-tagged gp130 (gp130-his) at 2 μg/mL in phosphate-buffered saline (PBS) (cat# 20012–027; Life Technologies, Carlsbad, CA) were coated directly onto each quadrant of a 384-well ELISA plate (cat# 464718; Thermo Scientific) and incubated at 4°C overnight. The antigen was aspirated, and wells were blocked with 3% nonfat milk (cat# 170–6404; Bio-Rad Laboratories, Hercules, CA) + 0.1% Tween 20 (cat# 251–07; J. T. Baker Chemical Co., Phillipsburg, NJ) in PBS. Mice sera or hybridoma supernatants were then applied directly to the plates and incubated at room temperature (22°C) for 1 h. IL-21–specific hybridomas were detected by using horseradish peroxidase (HRP)–labeled rabbit anti-mouse Fc **γ-**specific antibody (cat# 315-035-046; Jackson ImmunoResearch, West Grove, PA) at a dilution of 1: 10l,000. Plates were washed and developed by using SureBlue tetramethylbenzidine substrate (cat# 53-00-03; KPL, Gaithersburg, MD), followed by stop solution. The absorbance was read at 450 nm on an Envision plate reader (PerkinElmer, Waltham, MA). All hybridomas that bound human and cyno IL-21 were moved forward for further characterization.

Ligand-receptor (IL-21/IL-21R-Fc) competition ELISA was also used throughout the hybridoma generation campaign, initially to identify mice that elicited the best serum titers after IL-21 immunization and subsequently to identify the best neutralizing mAbs isolated after ELISA screening. Briefly, ELISA plates were coated with 2 μg of IL-21R-Fc per mL in PBS and incubated at 4°C overnight. The plates were blocked with 3% BSA (cat# A4503; Sigma-Aldrich, St. Louis, MO) + 0.1% Tween 20 in PBS at room temperature for 1 h. In parallel, test bleeds were serially diluted (to screen for highest responders) or IgG concentrations of hybridoma culture supernatants were normalized and preincubated with 2-nM biotinylated IL-21. After preincubation, the mixture was added to ELISA plates and incubated at room temperature for 30 min. The bound IL-21 was detected by using NeutrAvidin Protein, HRP (cat# 31001; Thermo Scientific), followed by the addition of tetramethylbenzidine and stop solution. The absorbance was read at 450 nm on an Envision plate reader (PerkinElmer). The samples were tested in duplicate, and data were analyzed using GraphPad Prism software. Binding of IL-21 to the immobilized IL-21R-Fc without inhibitors was measured as the total binding (100%). At each inhibitory antibody concentration, the percentage of inhibition was calculated as (1 –sample binding/total binding) × 100%.

### Mouse immunization and hybridoma selection

Animal welfare: Mice were housed in ventilated microisolator caging with water supplied by an automatic watering system. Feed was supplied ad libitum on a wire feeder. The caging included an enrichment loft and nesting material. The animal room was maintained at 74 +/-2 degrees F.

At the time these animal studies were performed the IACUC considered orbital sinus blood collection in mice a procedure that caused momentary pain or distress (USDA Pain Category C) and anesthetics were not required. The technical staff performing these procedures were highly skilled and could complete the process in conscious animals in under 10 seconds. The animals exhibited no signs of pain or distress after the procedure was completed and animals were monitored for 5 minutes after the procedure to confirm no adverse effects were noted.

Mice were euthanized via CO2 asphyxiation in a chamber using an appropriately regulated flow meter. As a secondary method of death, animals remained in the chamber for 5 minutes to confirm cessation of breathing.

Recombinant human and cyno IL-21 were conjugated to a carrier protein (BSA) with the Maleimide-Activated BSA Kit (cat# 77667; Thermo Scientific) to generate human IL-21–BSA and cyno IL-21–BSA immunogens. Immunization lasted 29 days at intervals of 3 to 4 days. Six-week-old female Balb/c mice received 8 rounds of intraperitoneal and metatarsal injections of human IL-21–BSA alternating with cyno IL-21–BSA in the dual-antigen protocol or human IL-21–BSA in the single-antigen protocol. Briefly, at each time point mice received 10 μg via intraperitoneal injection and 2.5 μg via metatarsal injection of immunogen emulsified in Imject Alum adjuvant (cat# 77161; Thermo Scientific). Adjuvants are known to play a crucial role in immunomodulating humoral responses and promoting the recruitment of T-helper cells. Alum was preferred over Freund adjuvant because the former is known to induce IL-4 [54, 55]. Retro-orbital bleeds were collected on days 0, 15, and 28 in serum separation tubes, and sera were tested by direct and competition ELISA. Mice displaying the highest neutralizing titers were selected to receive a prefusion boost of 10 μg of human IL-21–BSA, and 2 mice from each group were sacrificed on day 32. Splenocytes and lymph node cells were harvested and fused to P3-X63-Ag8.653 myeloma cells (ATCC CRL1580). IL-21–specific hybridomas were screened in a direct-binding ELISA. Human and cyno IL-21 cross-reactive supernatants were tested in a ligand-receptor competition assay as described above. Hybridomas exhibiting the best neutralization profile were moved into limited dilution cloning, and the screening assay cascade was repeated to confirm activity of the subclones.

### Phosphorylation of STAT3 signaling assay

Recombinant IL-2, IL-4, IL-7, IL-15, and IL-21 cytokines were each preincubated with anti–IL-21 antibodies in 96-well round-bottom plates (cat# 353072; Corning, Corning, NY) for 60 min at 37°C. After this incubation, PBMCs (0.4 × 10^6^ per well) were quickly added to each well and incubated with the mixture for 15 min at 37°C. Stimulation with human and cyno IL-21 induced a 3- to 5-fold increase in phosphorylated (p)STAT3 levels in total human PBMCs. After stimulation, the cells were fixed for 10 min (at 37°C) with prewarmed Lyse/Fix Buffer (cat# 558049; BD Biosciences), followed by permeabilization on ice for 30 min in Perm Buffer III (cat# 558050; BD Biosciences). Cells were washed twice and stained with phosphatidylethanolamine (PE)-conjugated anti-pSTAT3 (pY705, cat# 612569; BD Biosciences), PE-conjugated anti-pSTAT5 (pY694, cat# 612567; BD Biosciences) or PE-conjugated anti-pSTAT6 (pY641, cat# 562078; BD Biosciences) for 30 min at room temperature. Cells were washed with stain buffer (PBS–2% fetal calf serum) and analyzed on an LSR II flow cytometer (BD Biosciences), using FACSDiva software. To calculate percent inhibition, the ratio of pSTAT3 levels (MFI) is calculated between cells stimulated in the presence or absence of MEDI7169 after basal levels of pSTAT3 (level in unstimulated cells) are subtracted from each value. Therefore, differences in basal levels of pSTAT3 from donor to donor do not influence the inhibition observed in this assay. Differences in the lower level of inhibition observed in this assay represent a non-specific effect of IgG in this assay.

### PC differentiation assay with recombinant human IL-21

B cells were cultured in 96-well round-bottom plates (cat# 353072; Corning) at a density of 0.5 to 1.0 × 10^5^ cells per well in a final volume of 150 μL of complete B-cell medium as previously described [34] Cells were stimulated with a combination of recombinant IL-21 (30 ng/mL), anti-CD40 (0.1 μg/mL), and anti-IgM F(abʹ)2 (5.0 μg/mL). IL-21 antibody was added at the initiation of the culture period at a final concentration of 0.003 to 10 μg/mL. B cells were cultured for 7 days. After 7 days of stimulation, B cells were stained for 30 min at 4°C with an antibody cocktail specific for PCs. PCs were labeled with PerCP Cy5.5–conjugated anti-human CD19 (cat# 340951; BD Biosciences), activated protein C (APC)–conjugated anti-human CD38 (cat# 340439; BD Biosciences), and PE-conjugated anti-human IgD (cat# 555779; BD Biosciences). Samples were washed, and AccuCount Particles (cat# ACBP-100-10; Spherotech, Lake Forest, IL) were added to all wells for enumeration of PCs. Cells were run on an LSR II flow cytometer (BD Biosciences) using FACSDiva software, and samples were analyzed with FlowJo software (version 9).

### Co-culture of B cells and activated CD4^+^ T cells

Prior to culture, CD4^+^ T cells were treated with mitomycin C as previously described [35] Briefly, cells were incubated with mitomycin C (30 μg/mL) for 30 min at 37°C, then washed twice and rested at 37°C for an additional 30 min. A total of 1.0 × 10^5^ mitomycin C–treated CD4^+^ T cells and 0.5 × 10^5^ purified B cells (per well) were co-cultured with anti-CD3/anti-CD28 beads (cat# 111.31D; Life Technologies). Anti-CD3/anti-CD28–coated beads were used to stimulate CD4^+^ T cells in a 5:1 T-cell–to–bead ratio. IL-21 antibody was added at the initiation of the culture period at a final concentration of 0.01 to 30 μg/mL. B cells were cultured with activated CD4^+^ T cells for 7 days. On day 7, co-cultured cells were processed for fluorescence-activated cell sorting and analyzed as described above. To identify PCs, cells were stained with an antibody cocktail of fluorescein isothiocyanate–conjugated anti-human CD4 (cat# 317408; BioLegend, San Diego, CA), PerCP Cy5.5–conjugated anti-human CD19, APC-–conjugated anti-human CD38, and PE-conjugated anti-human IgD.

### Naive CD4^+^ T-cell expansion assay

Naive CD4^+^ T cells were cultured in 96-well flat-bottom plates (cat# 353072; Corning) at a density of 0.1 × 10^6^ cells per well in a final volume of 200 μL of complete T-cell medium (Medimmune). Cells were stimulated with a combination of recombinant human IL-21 (25 ng/mL) and anti-CD3 Dynabeads (0.1 × 10^6^ beads per well). IL-21 antibody was added at the initiation of the culture period at a final concentration of 0.001 to 30 μg/mL. T cells were cultured for 4 days. T-cell expansion was quantified by measuring ATP after 4 days of stimulation, using the Cell Titer-Glo Luminescent Assay (cat# G7570; Promega, Madison, WI) according to the manufacturer’s instructions.

### Molecular cloning of the variable domains from the hybridomas

Messenger RNA from clones that went through limited dilution cloning was isolated using the Dynabeads mRNA Direct Kit (cat# 61012; Invitrogen, Carlsbad, CA) according to the manufacturer’s instructions. Complementary DNA (cDNA) was synthesized by reverse transcriptase polymerase chain reaction (PCR), using SuperScript III Reverse Transcriptase (cat# 18080044; Invitrogen), and cDNAs were used as templates to amplify antibody-variable V_L_ and V_H_ regions. PCR was performed with high-fidelity Taq polymerase (cat# 11304011; Invitrogen) and the Novagen Mouse Ig Primer Set (cat# 69831–3; VWR, Radnor, PA), which included 6 V_H_ and 7 V_L_ primer sets. Amplified V_L_ and V_H_ PCR products were purified and cloned into pCR 2.1 Topo vectors (cat# K450002; Invitrogen), and colonies containing V_H_ or V_L_ inserts were picked for PCR amplification and sequenced.

### Humanization of murine anti–IL-21 antibody

CDR grafting combined with a rational design strategy was employed for humanization. The sequences of the V_H_ and V_L_ of murine mAb 19E3 were compared with the human antibody germline sequences available in the public National Center for Biotechnology Information database (https://www.ncbi.nlm.nih.gov/igblast) [56]. The human germline sequences with highest homology for each FR of the murine mAb were selected as template. The human germline template chosen for the V_L_ was a combination of O14 (FR1), O18 (FR2), L23 (FR3), and JK1 (FR4) for the optimum matches. One germline family, V_H_1-46, was identified as the acceptor FR for V_H_ because it had the best matches for each of the three FRs. The J-segment genes were compared with the parental sequences over D-J segments, and JH4 was selected for the V_H_ template. The antibody Kabat numbering and Chothia canonical structure class were determined by using the online tool (http://www.bioinf.org.uk/abs/) provided by Dr. Andrew C.R. Martin’s group. Critical murine FR residues, including Vernier zone, canonical class residues, and V_H_/V_L_ interface residues, that could have an impact on CDR loop structure and thereby affect antigen binding [40–42] were identified and analyzed for design of back-mutations onto the human FR templates. CDR residues were fused into the designed human FRs for both V_H_ and V_L_ for generating the humanized antibody. The V_H_ and V_L_ genes of humanized versions of 19E3 [57] were synthesized by GeneART (Life Technologies) and then cloned into a pOE IgG1 expression vector (MedImmune) and expressed and purified as described below.

### Antibody engineering, cloning, expression, and purification

Oligonucleotide-directed mutagenesis was used to incorporate amino acid sequence changes in V_L_ or V_H_ genes by overlapping PCR and then cloned into a pOE IgG1 expression vector. Antibody variants were expressed transiently in HEK293F cells (Life Technologies) as full-length IgG1 kappa molecules according to the manufacturer’s instructions. After expression for 7 days, conditioned medium was filtered through a 0.2-μm filter and loaded on a HiTrap Protein A HP column (cat# 17040303; GE Healthcare, Little Chalfont, UK); washed with 10-column volumes of PBS, pH 7.4; and eluted with IgG Elution Buffer (cat# PI211004; Thermo Scientific), followed by buffer exchange to PBS. The quality and monomer content were evaluated by analytical size-exclusion high-performance liquid chromatography and sodium dodecyl sulfate–polyacrylamide gel electrophoresis.

### HTRF antibody epitope competition assay

The HTRF antibody epitope competition assay measures the ability of unlabeled antibodies to compete with a labeled parent antibody for binding to a target antigen [44] It can be used to rank the affinity of antibodies that bind to the same epitope. The parental antibody 19E3 was labeled with *N*-hydroxysuccinimide–activated europium cryptate, using an HTRF labeling kit (cat# 62EUSPEA; Cisbio, Codolet, France) according to the manufacturer’s instructions. The assay was performed by mixing unlabeled antibodies at various concentrations with 0.5 nM biotinylated IL-21 in PBS buffer, pH 7.2; 0.1% BSA; and 0.4 M potassium fluoride (cat# 103444T; VWR), followed by the addition of 1.2 nM europium cryptate–labeled parental antibody 19E3 and 0.5 nM streptavidin–XL665 (cat# 611SAXLB; Cisbio) in 384-well Greiner plates (cat# M6186; Sigma-Aldrich) (total volume, 20 μL per well). The fluorescence was measured on a PerkinElmer Envision plate reader. The 665/620-nm emission ratio was used for data analysis. The samples were tested in duplicate, and IC_50_s were calculated using GraphPad Prism software.

### Capillary isoelectric focusing immunoassay

Antibody samples were analyzed on a NanoPro 1000 Instrument (ProteinSimple) according to the manufacturer’s instructions. Unless otherwise indicated, all reagents were obtained from ProteinSimple. Briefly, mAbs were diluted to 4 μg/mL in Simple Dilute (cat# 040–649). The samples were then mixed with Premix G2 pH separation gradient containing fluorescence-labeled pI standards (pH 5.5, cat# 040–028; pH 7.3, cat# 040–032; pH 8.4, cat# 040–036; pH 9.7, cat# 040–790); 0.25% tetramethylethylenediamine (cat# 0761; Amresco, Solon, OH); 50% Premix G2, pH 4–9 (cat# 040–969); and 4% pH 8 to 10.5 Pharmalytes (cat# 17-0455-01; GE Healthcare). The detection anti-human IgG Fc-HRP–conjugated mouse antibody (cat# 209-035-098; Jackson ImmunoResearch) was diluted 1:200 into Antibody Diluent (cat# 040–309). Luminol (cat# 040–652) and Peroxide-XDR (cat# 041–084) were mixed in a 1:1 ratio and used as a chemiluminescent substrate. The samples (12 μL per well) and detection reagents (20 μL per well) were loaded into a 384-well assay plate (cat# 040–663; ProteinSimple). The 384-well sample plate, charge separation capillary box (cat# CBS 701; Protein Simple), and buffers (cat# 040–337, 040–338, and 041–108; ProteinSimple) were loaded into a NanoPro 1000 instrument according to the manufacturer’s instructions. The automated separation and detection procedures were performed using the default charge separation settings. The capillaries were imaged and analyzed with Compass software (ProteinSimple).

### Epitope binning

Epitope binning was assessed by using an Octet 384 instrument (Pall ForteBio) as described previously [58], with some modifications. Briefly, biotinylated human IL-21 at 0.5 μg/mL in PBS, pH 7.2; 3-mg/mL BSA; and 0.05% (vol/vol) Tween 20 were captured on streptavidin biosensors (cat# 18–5019; Pall ForteBio) for 250 s. The IL-21–coated tips were each dipped into a different “saturating” IL-21 mAb (300 nM) for 300 s. After a wash of unbound protein in a buffer blank well, sensor tips were moved into wells containing a mixture of the “competing” IL-21 mAb with the saturating IL-21 mAb. If the saturating mAb and the competing mAb bind to the same epitope on IL-21, no additional mass shift will be detected; if the two test mAbs do not block each other’s binding to IL-21, an increased shift in mass will be detected. The same array of saturating IL-21 mAb was measured against three different competing mAbs. Competition studies using IL-21R-Fc as a comparator were set up in the same manner.

### Affinity measurement

The equilibrium dissociation constants (K_D_) for mAbs against IL-21 were measured using a kinetic exclusion assay (KinExA 3000; Sapidyne) platform. Briefly, separate 50-mg quantities of Azlactone beads (cat# 53111; Thermo Scientific) were coated with human IL-21 at 5, 75, or 150 μg/mL according to the manufacturer’s instructions. Batch volumes of mAbs were prepared at 500 fM and 50 pM in sample buffer (10 mM *N*-2-hydroxyethylpiperazine ethanesulfonic acid [HEPES], pH 7.4; 0.1% BSA; 300 mM NaCl; 5 mM CaCl_2_; and 0.02% NaN_3_); each was dispensed into 2 separate sets of 13 tubes. Human IL-21 was added into 1 tube from each series at a concentration of either 4 nM (50-pM mAb series) or 100 pM (500-fM mAb series), then serially diluted across 11 of the remaining tubes (for a final IL-21 concentration of 1.95 fM to 100 pM for the 500-fM mAb series and 78.1 fM to 4 nM for the 50-pM mAb series), leaving 1 sample tube in each series as the mAb-only control. The mixtures were equilibrated at room temperature for 2 to 7 days and analyzed on the KinExA 3000 platform. DyLight 649–labeled, goat–anti-human IgG (heavy and light chain) (cat# 109-495-068; Jackson ImmunoResearch) secondary reagent was passed over the beads to detect mAb bound to the IL-21–coated beads. A binding isotherm was generated for each series to plot the amount of free mAb detected. The resulting two binding curves were evaluated in a dual-curve analysis, using the instrument’s evaluation software.

## Supporting information

S1 Fig Differential binding of IL-21 to IL-21 receptors. (A) Human and (B) cyno IL-21R-Fc was coated on an ELISA plate. Serial dilutions of biotin-labeled human and cyno IL-21 were added. Bound IL-21 was detected by streptavidin-HRP. OD = optical density.
(TIF)

S2 Fig Comparison of human and cyno IL-21 sequence on IL-21/IL-21R co-crystal structure. (A) Pairwise Alignment of human and cyno IL-21 was performed using the Pearson-Lipman method. Human and cyno IL-21 show 95.5% identity. One amino acid variation, V91L (red frame), occurs in the receptor binding region. (B) Amino acid variations between human and cyno IL-21 were highlighted (red) on an available crystal structure for human IL-21/IL-21R. Five of the six variations occur at sites distal to the receptor/cytokine interface.
(TIF)
