# Supplementary figures and images for: Discovery and characterization of potent IL-21 neutralizing antibodies via a novel alternating antigen immunization and humanization strategy
Source: PLoS One. 2019 Jan 25;14(1):e0211236. doi: 10.1371/journal.pone.0211236 (PMC6347146; doi:10.1371/journal.pone.0211236)

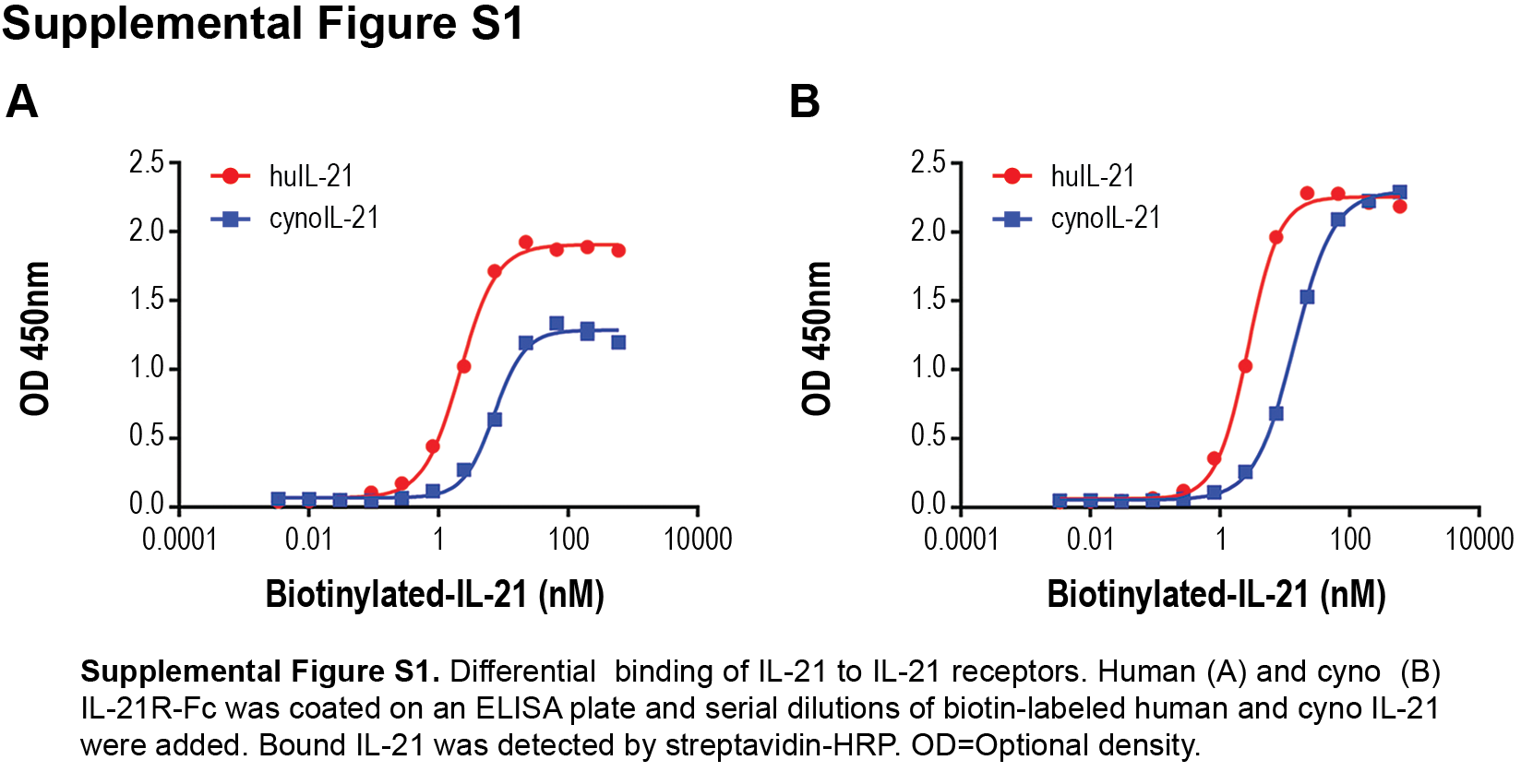

Supplement: S1 Fig — (A) Human and (B) cyno IL-21R-Fc was coated on an ELISA plate. Serial dilutions of biotin-labeled human and cyno IL-21 were added. Bound IL-21 was detected by streptavidin-HRP. OD = optical density. (TIF) [file pone.0211236.s001.tif]

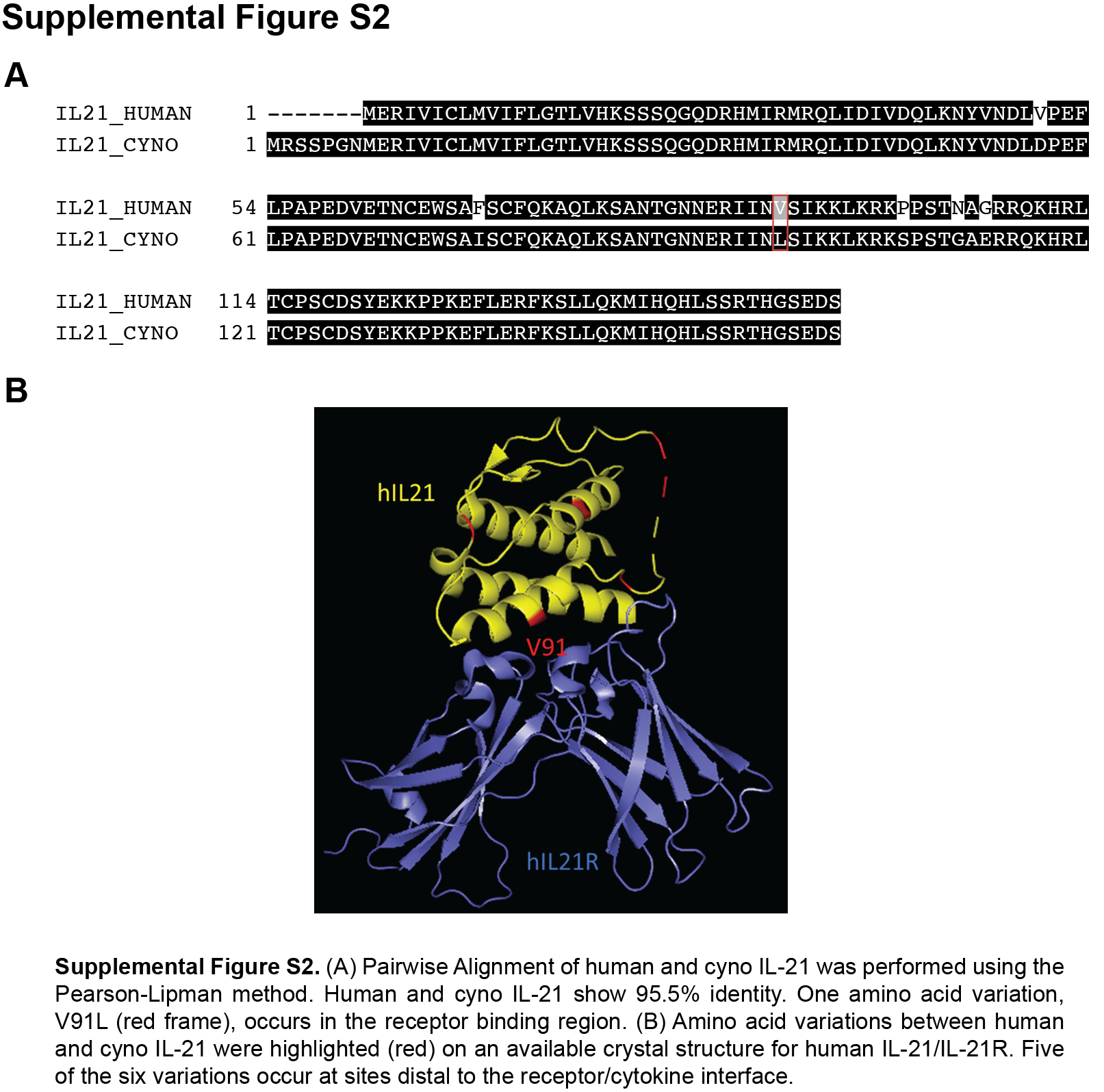

Supplement: S2 Fig — (A) Pairwise Alignment of human and cyno IL-21 was performed using the Pearson-Lipman method. Human and cyno IL-21 show 95.5% identity. One amino acid variation, V91L (red frame), occurs in the receptor binding region. (B) Amino acid variations between human and cyno IL-21 were highlighted (red) on an available crystal structure for human IL-21/IL-21R. Five of the six variations occur at sites distal to the receptor/cytokine interface. (TIF) [file pone.0211236.s002.tif]
